# Parasite load as a marker of pathogenicity in *Dientamoeba fragilis* infections

**DOI:** 10.1097/MD.0000000000041963

**Published:** 2025-03-28

**Authors:** Ander Burgaña Agoües, Rosa Abellana Sangra, Mónica Ballestero-Téllez, Mireia Rajadell-Guiu, Marc Garreta-Esteban, Tomàs M. Perez-Porcuna

**Affiliations:** a Atenció Primària, Fundació Assitencial Mútua Terrassa, Terrassa. Spain. Fundació Docència i Recerca Mútua de Terrassa, Terrassa, Spain; b Departament Fonaments Clínics, Universitat de Barcelona, Barcelona, Spain; c Centre Analítiques Terrassa, Catlab AIE, Clinical Microbiology Department, Terrassa, Spain.

**Keywords:** carrier state, *Dientamoeba*, family health, leukocyte L1 antigen complex, parasite load, virulence

## Abstract

*Dientamoeba fragilis* is a globally widespread intestinal parasite and one of the most frequent in humans, often leading to primary care consultations. The pathogenic potential of this parasite remains unclear due to limited evidence and contradictory study results. This study investigated the pathogenicity of *D fragilis*, focusing on the critical knowledge gap regarding the relationship between parasite load and associated symptomatology. A prospective case-control study matched by household unit was conducted, considering individuals with gastrointestinal symptoms and *D fragilis* in stool as cases, and their asymptomatic household members with *D fragilis* in stools as controls. *D fragilis* detection was performed in parallel using light microscopy and real time-polymerase chain reaction. The study was carried out in 7 primary care centers over 12 months. Parasite load was measured as the number of trophozoites per field at a magnification of 40x by microscopy and by cycle threshold values in RT-PCR. A total of 218 individuals were recruited: 74 symptomatic cases and 144 household members, of whom 57 (39.6%) were *D fragilis*-positive asymptomatic controls. The proportion of individuals with *D fragilis* and a parasite load less than 1 trophozoite per field was higher in asymptomatic individuals (controls) than in symptomatic cases (47.7% vs 3.1%, respectively) (*P *< .001). Parasite load is associated with the presence of gastrointestinal symptoms, supporting the pathogenicity of *D fragilis*. Any diagnostic approach for *D fragilis* should incorporate or be complemented by quantitative information to accurately estimate parasite load and enhance treatment decision-making.

## 1. Introduction

Gastrointestinal symptoms are a common reason for primary care consultations, with abdominal pain or diarrhea accounting for up to 10% of visits.^[1,2]^ Among infectious causes, protozoa such as *D fragilis* are frequently identified. This non-flagellated protozoan parasitizes the human intestine, affecting populations worldwide, with a prevalence that significantly varies depending on the geographic region, diagnostic methods used (mainly light microscopy [LM] and real time polymerase chain reaction [RT-PCR]), and population characteristics. Globally, prevalence estimates range from 0.1% to 62%.^[3]^ In Spain, the prevalence ranges between 0.4% and 24% in children and 2% to 9% in adults.^[4]^ The sensitivity of RT-PCR is higher than that of LM, increasing the detection rate, especially in low-parasite loads, often with a weak correlation to symptoms.^[3,5,6]^

Humans are the primary hosts of *D fragilis* Until recently, trophozoites were the only known life cycle stage of *D fragilis* in humans, which deteriorate rapidly after being excreted in stool. Several theories have been proposed to explain how *D fragilis* could survive outside the host for a short period of time to facilitate transmission. One possibility is that *D fragilis* is transmitted via a helminth vector or through a resistant pseudocyst or cyst stage.^[7–11]^

The pathogenicity of *D fragilis* has been debated for years, with conflicting evidence about its role in causing gastrointestinal symptoms.^[12–17]^ Some studies have described symptom improvement after treatment while others have not found a clear relation between parasite colonization and symptoms.^[16–21]^

The only study linking parasite load to pathogenicity was conducted by El Gayar et al using an animal model, in which they demonstrated that an infectious dose of *D fragilis* is necessary to cause colitis and active inflammation.^[22]^ A mouse model fulfilling Koch’s postulates has also shown that cysts present in rodent feces can cause disease. Infected mice exhibited weight loss, mild intestinal inflammatory response, increased fecal calprotectin (f-CP), and improvement after treatment.^[7]^

In 2019, Brands et al^[19]^ analyzed whether symptoms were related to the presence of *D fragilis* in feces, symptom resolution after parasite eradication, and its consideration as a pathogen using LM and RT-PCR. They concluded that there was insufficient evidence to clarify the pathogenicity of *D fragili* due to contradictory findings. In 2020, Van Kalleveen et al systematically reviewed diagnostic considerations and treatment efficacy in children with *D fragilis*, highlighting diagnostic complexity due to symptom variability and the lack of standardized and sensitive methods. They concluded that RT-PCR might be more sensitive than LM examination and observed variability in the efficacy of treatments, such as metronidazole and paromomycin, emphasizing the need for more research on the best therapeutic option.^[6]^ The discrepancies among previous scientific studies regarding the relationship between diagnosis, symptomatology, and treatment response did not take into account the diagnostic method employed or the parasite load.

The clinical presentation of *D fragilis* infection ranges from asymptomatic cases to nonspecific digestive symptoms, with abdominal pain and diarrhea being the most common. However, abdominal distension, nausea, and vomiting have also been reported. Diagnostic is initially based on the assessment of these early symptoms and is confirmed through a stool parasitological examination, provided that no other underlying cause can explain the clinical presentation.^[23]^ Transmission studies show intrafamilial spread rates between 30% and 50%.^[24,25]^ The use of the inflammatory marker f-CP could support pathogenicity, although its relationship with *D fragilis* is underexplored.^[26]^ Variations in pathogenicity may be due to differences in gene expression, such as cysteine proteases and other virulence factors.^[27]^

Most therapeutic guidelines indicate that *D fragilis* often does not require treatment due to its asymptomatic nature. However, if symptoms persist and no other cause is identified, paromomycin, clioquinol, and metronidazole are recommended as the drugs of choice.^[3]^

The diagnosis of *D fragilis* by LM is highly challenging, requiring expertise in parasitology and significant time investment, which often results in underdiagnosis. Additionally, the uncertain clinical significance of this parasite has led to it being overlooked by many laboratories and being frequently classified as a commensal organism, further limiting the availability of clinical data on *D fragilis*.^[12]^ The introduction of molecular techniques, particularly multi-target assays for parasites, has improved its detection during syndromic investigations of parasitic infections. However, the lack of clinically relevant information linking molecular findings to actual clinical presentations remains a significant gap in this field.

*D fragilis* is a globally distributed pathogen with a high prevalence in individuals presenting with gastrointestinal symptoms, making it a frequent concern in primary care settings. This study aimed to investigate the parasitological, individual, and epidemiological factors associated with the pathogenicity of *D fragilis*, underscoring its clinical relevance in routine medical practice. Specifically, this study addresses a critical knowledge gap by examining the relationship between the parasite load of *D fragilis* and its association with symptomatology.

## 2. Materials and methods

### 2.1. Design

We conducted a prospective, observational, analytical case-control study matched by household unit in a population with *D fragilis* to investigate factors associated with the clinical symptoms of intestinal infection by these parasites. The study population consisted of ambulatory patients from the primary care area of Mútua de Terrassa (approximately 290,000 people) in Terrassa, Spain, from December 2022 to December 2023.

Cases were defined as patients with *D fragilis* (intestinal infection) and compatible clinical symptoms (presence of abdominal pain, diarrhea, anal itching, or other gastrointestinal symptoms), while controls were household members living with the cases who had *D fragilis* (intestinal infection) but no clinical symptoms. The household unit was defined as the group of people residing in the same domicile. All individuals in the study received treatment according to their diagnosis.

All incident cases and their household members who agreed to participate were included. Patients treated with antibiotics, those with parasitic, bacterial, or viral coinfections, patients diagnosed with celiac disease, inflammatory bowel disease, intestinal ulcers, or other gastrointestinal disorders that could explain the symptoms, and patients unable to undergo the required tests and/or visits during the study follow-up were excluded.

Participants were recruited from 7 primary care centers (CAP Sant Cugat, CAP Valldoreix, CAP Turó de Can Matas, CAP Rubí, CAP Terrassa Sur, CAP Oest, CAP Rambla). The sample selection was made up of incident cases diagnosed with intention to treat detected in primary care centers and their household members. Patients with gastrointestinal symptoms in whom the presence of *D fragilis* in stool samples was detected by LM (in at least one of three stool samples) within the previous 2 months were invited to participate as cases. Asymptomatic household members were also invited to participate. Cases and controls were matched by household unit to control for potential biases related to the household environment.

**Figure 1.**
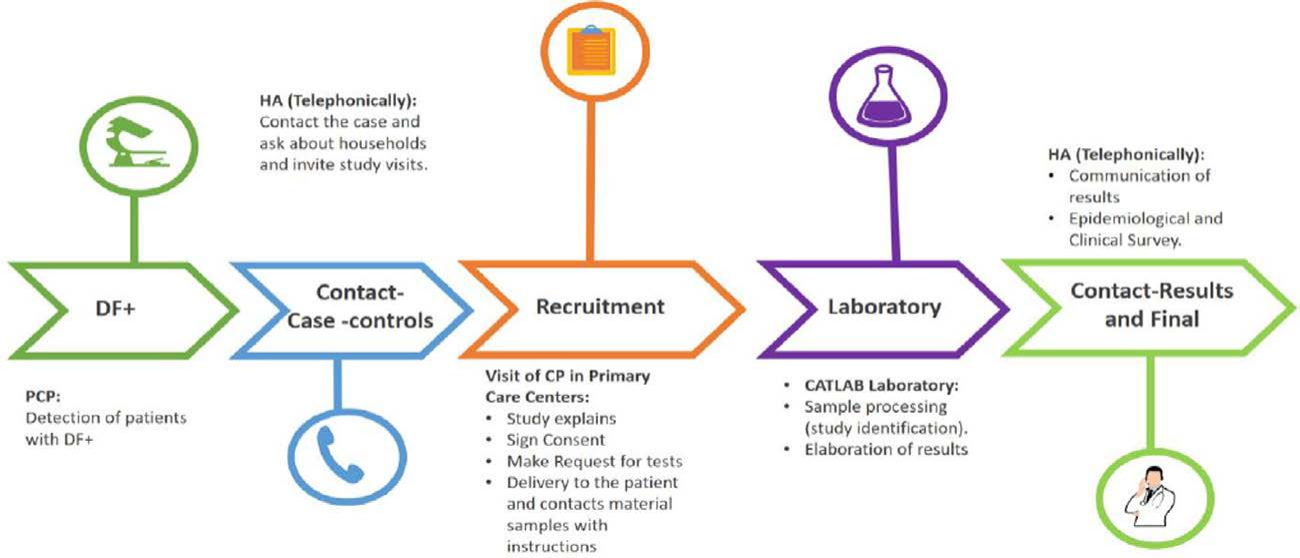
Study diagram. CP = collaborating physician, DF+ = *Dientamoeba fragilis* infection, HA = healthcare administrator, PCP = primary care physician.

The main study variables were parasite load in fresh stool samples according to LM and f-CP. Sociodemographic variables were collected using an epidemiological survey (age, sex, number of household members), and clinical variables with a clinical survey (presence of chronic diseases). Participants were asked about symptoms, including abdominal pain (any pain located in the abdominal area regardless of duration, intensity, and location), diarrhea (an increase in the frequency of normal bowel movements (3 or more per day) and/or decreased consistency, either continuously or intermittently), or anal itching. Results were communicated by phone, and symptomatic participants were administered a symptom survey (Fig. 1).

### 2.2. Laboratory methods

Three stool samples were collected from each case and their household members. The first sample was collected using a swab with liquid Cary-Blair medium (DeltaSwab Cary Blair, Deltalab Spain) for coproculture and molecular diagnosis of enteropathogens and parasites. The second stool sample was collected in a sterile container without preservative medium for the diagnosis of rotavirus and adenovirus by immunochromatography and f-CP determination. Lastly, the third sample consisted of three consecutive daily stool samples placed in specific parasite transport medium (Formol-Ether 10%, Heipha Diagnostika, Eppelheim, Germany) for detection of intestinal parasites (Mini Parasept® SF, Apacor, Wokingham, United Kingdom). The stool cultures were performed following standard methodology using selective and enrichment media for enteropathogens.^[28]^

To rule out bacterial coinfections, molecular diagnosis of acute bacterial gastroenteritis was carried out using multiplex RT-PCR (Allplex™ GI-EB Screening Assay, Seegene Inc, Seoul, South Korea) which includes detection targets for *Campylobacter* spp., *Escherichia coli* and/or *Shiga* toxin-producing, *Shiga* (stx1/2), *E coli O157*, *Salmonella* spp., *Yersinia enterocolitica*, *Shigella* spp. and/or *enteroinvasive E. coli*. Molecular detection of parasites was conducted using multiplex RT-PCR (Allplex™ GI-Parasite Assay, Seegene Inc), following nucleic acid extraction as per the manufacturer’s instructions. This assay detects *D fragilis, Blastocystis hominis, Giardia lamblia, Entamoeba histolytica, Cryptosporidium* spp. and *Cyclospora cayetanensis*.

For viral coinfections, immunochromatography was performed for the diagnosis of rotavirus and adenovirus according to the manufacturer’s instructions (CerTest Rotavirus + Adenovirus, Spain) with results verified by 2 independent observers.

The Graham technique was employed for the collection of a single sample from the anal margins using transparent adhesive tape, which was then applied to a microscope slide for subsequent examination by LM.

For parasite detection, three consecutive parasite samples were processed using a concentration procedure by centrifugation (1500 rpm, 1 minute). After concentration, the supernatants from the three serial samples of the same patient were combined, and fresh preparations were evaluated by LM. The identification of *D fragilis* was based on morphology. All personnel performing sample observations underwent intensive training for accurate *D fragilis* detection and were assessed for result consistency through an evaluation of the correlation between independent observations.

When *D fragilis* was observed in a sample, a semiquantitative count of trophozoites was made. A minimum of 100 fields per preparation was screened using an 40× objective lens. The following scale was used: less than 1 trophozoite per field (a maximum of 1 trophozoite observed in any of 100 fields), 1 to 2 trophozoites per field (at least 1 trophozoite per field up to a maximum of 2 in any of 100 fields), and more than 2 per field. All *D fragilis* detected by LM were confirmed by RT-PCR, and the cycle threshold (Ct) value was recorded. f-CP was measured by turbidimetry using the Bühlmann fcal® turbo assay, reported in mg/kg.

### 2.3. Statistical analysis

Continuous variables were expressed as means and standard deviations (SD) and frequencies and percentages for categorical variables. For the association analysis, cases and controls were matched by household unit to control for confounding variables, improve the precision and validity of the results, and reduce bias. We used conditional logistic regression to investigate the relationship between the presence of symptomatology and demographic characteristics, parasite load levels, f-CP levels and Ct RT-PCR of *D fragilis*. The type I error rate was set at 5%. All data analyses were conducted using R (v.4.3.1). The associations were measured in terms of odds ratios (OR) and the 95% confidence interval (CI).

## 3. Results

**Figure 2.**
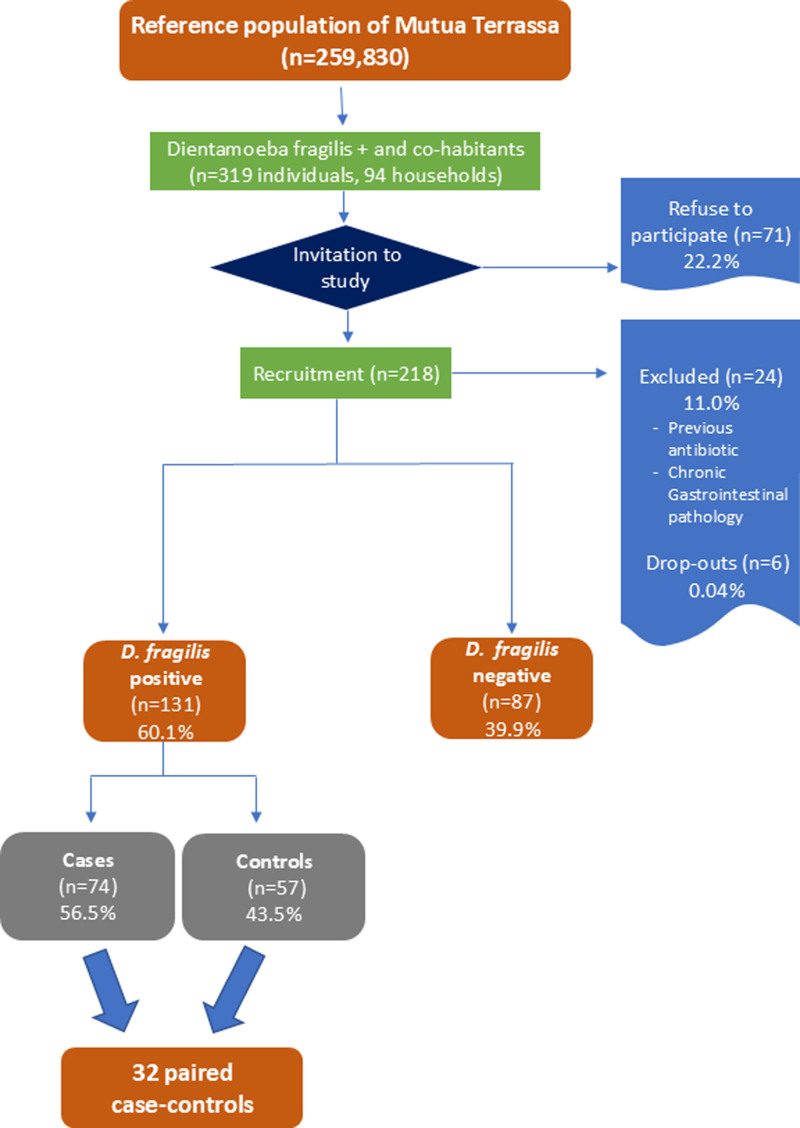
Flowchart of participant enrollment and inclusion.

A total of 94 individuals who presented with gastrointestinal symptoms were identified and were diagnosed with *D fragilis* in their stools, while other co-infections and comorbidities were ruled out. All of these indivudals and their household members (a total of 319 individuals) were invited to participate in the study. After excluding those who declined to participate (22.2%, 71/319) and those who were either excluded or withdrew from the study, a total of 218 individuals were ultimately recruited. Among these, *D fragilis* was not detected in 39.9% (87/218), while in the remaining 60.1% (131/218) it was. Among those with *D fragilis*, 74 (56.5%) were symptomatic patients (cases) and 57 (43.5%) were asymptomatic household members (controls). In the final analysis, 32 household units, in which at least one case and one control could be matched, were included (Fig. 2).

Regarding the clinical presentation of the 74 symptomatic individuals, a summary is provided in Table 1. In terms of ethnicity within the household units, 71.9% were Caucasian (23/32), 18.8% Hispanic or Latino (6/32), and 9.4% were of other ethnicities (3/32).

**Table 1:** Summary of clinical symptoms of cases.

	All cases	Cases included in paired analysis
	N = 74	N = 32
Asymptomatic	74 (100%)	32 (100%)
Abdominal pain		
No	24 (32.4%)	9 (28.1%)
Yes	50 (67.6%)	23 (71.9%)
Diarrhea		
No	43 (58.1%)	18 (56.2%)
Yes	31 (41.9%)	14 (43.8%)
Anal itching		
No	71 (95.9%)	32 (100%)
Yes	3 (4.05%)	0(0%)
Other symptoms		
No	71 (95.9%)	31 (96.9%)
Yes	3 (4.05%)	1 (3.12%)

### 3.1. Mean results

Among the 74 cases and their household units, 32 cases were matched with at least one positive asymptomatic control. There was a total of 57 matched controls. The rate of persons infected by family was 0.78 (SD 1.20).

Association analyses revealed that parasite load, measured by the number of trophozoites per field, and age were significantly associated with the presence of symptoms.

Regarding parasite load, the proportion of individuals with < 1 trophozoite per field was significantly higher among the controls (47.7%) compared to the cases (3.1%) (*P* < .001). It was also observed that the mean age among the cases was lower than that of the controls, being 17.9 years (SD = 19 years) versus 26.9 years (SD = 18.6). In contrast, the parasite load measured by RT-PCR Ct values for *D fragilis* shows no association with symptomatology, with PCR Ct values of 33.7 cycles (SD = 4.92) in cases and 35.0 cycles (SD = 4.35) in controls (*P* = .529) (Table 2).

**Table 2:** Comparison of paired positive cases and their infected household controls.

	[ALL]	Case	Control	*P* value	N	Odds ratio (95% confidence interval)
	N = 89	N = 32	N = 57			
Gender				.275	89	
Female	41 (46.1%)	17 (53.1%)	24 (42.1%)			
Male	48 (53.9%)	15 (46.9%)	33 (57.9%)			1.64 (0.67, 3.99)
Age (years)	23.6 (19.1)*	17.9 (19.0)*	26.9 (18.6)*	.012	89	0.96 (0.93, 0.99)
Fecal calprotectin (mg/kg, categorized)				.072	88	
≤50 mg/kg	27 (30.7%)	10 (32.3%)	17 (29.8%)			1
50–200 mg/kg	53 (60.2%)	16 (51.6%)	37 (64.9%)			0.51 (0.16, 1.62)
≥200 mg/kg	8 (9.09%)	5 (16.1%)	3 (5.26%)			5.23 (0.54, 50.27)
Parasite load *D fragilis* (trophozoites × field)				.012	89	
<1 × field	28 (31.5%)	1 (3.12%)	27 (47.4%)			1
1–2 × field	49 (55.1%)	26 (81.2%)	23 (40.4%)			18.33 (2.53, 142.7)
>2 × field	12 (13.5%)	5 (15.6%)	7 (12.3%)			14.18 (1.47, 137.1)
Threshold cycle RT-PCR of *D fragilis* (cycles)	34.6 (4.53)*	33.7 (4.92)*	35.0 (4.35)*	.529	63	0.94 (0.78, 1.13)

RT-PCR = real time-protein chain reaction.

* Standard deviation.

Other variables, such as f-CP levels and gender, did not show statistically significant associations with the presence of symptoms.

In a multivariate model, parasite load was directly associated with symptomatology. The adjusted OR (aOR) for 1 to 2 trophozoites and > 2 trophozoites per field was 31.57 (95% CI: 3.12, 319.2) and 15.13 (95% CI: 1.27, 180.9), respectively. Age was inversely related to symptomatology, with an aOR of 0.94 (95% CI: 0.90, 0.98), indicating that the likelihood of symptom presentation decreases with increasing age (Table 3).

**Table 3:** Multivariate analysis of age and parasite load of *Dientamoeba fragilis.*

Variable	OR	95% CI	*P* value
Parasite load *D fragilis* (trophozoites × field)			
<1 × field	–	–	
1–2 × field	31.57	3.12, 319.2	.001
>2 × field	15.13	1.27, 180.9	.016
Age (years)	0.94	0.90, 0.98	.011

CI = confidence interval, OR = odds ratio.

## 4. Discussion

This is the first human study linking the parasite load of *D fragilis* with gastrointestinal symptoms, supporting its pathogenic potential as suggested by animal models.^[22]^ Our findings underscore the importance of parasite load for diagnosing *D fragilis* infections, especially in symptomatic cases. Unlike molecular diagnostic methods, LM-based parasite load quantification proved a valuable tool for assessing pathogenicity.

This study demonstrates that the symptomatology of *D fragilis* infections depends, at least in par, on the parasite load harbored by the host. The findings of the present study help explain the disparity in the results of previous studies regarding the diagnosis of *D fragilis*, the symptomatology of the infection, and response to treatment. Furthermore, our results underscore the importance of evaluating parasite load to potentially define the clinical status of individuals infected with *D fragilis*, whether as asymptomatic carriers or as cases of *D fragilis*-associated disease. Moreover, these findings highlight the need for the quantification of parasite load when diagnosis is performed via LM, similar to the routine quantification employed for other parasites that may be part of the commensal microbiota, such as *B hominis*. Given that *D fragilis* infections are a common consultation in primary care, our study highlights the relevance of understanding their clinical significance in this context.

Control group consisting of asymptomatic household members, also infected with *D fragilis*, allowed minimizing possible bias. By matching cases and controls from the same household, we controlled for environmental and genetic factors, providing stronger evidence that parasite load, rather than other confounding variables, contributes to the presentation of symptoms. This design marks an improvement over previous studies, which often used unmatched controls or convenience sampling, leading to less reliable results.^[16,19–21]^

Despite decades of debate surrounding the pathogenicity of *D fragilis*, our findings help to clarify this ongoing discussion. Previous studies have produced mixed results. with some showing symptom resolution following treatment,^[21]^ while others reported no clear correlation between the presence of this parasite and symptoms.^[16–19]^ Our study, however, demonstrates that individuals with higher parasite loads are significantly more likely to present gastrointestinal symptoms, supporting the notion that *D fragilis* can be pathogenic.

The discrepancy among studies may stem from variations in diagnostic methods. PCR, with its high sensitivity, detects even low levels of *D fragilis*, often leading to the identification of asymptomatic carriers.^[3]^ In contrast, LM-based parasite load quantification provides a more accurate picture of parasite burden, which is more closely associated with the presence of symptoms. Our study showed that individuals with less than one trophozoite per field were far more likely to be asymptomatic, while higher parasite counts were strongly linked to symptomatology.

The potential pathogenicity of *D fragilis* could also be strain-dependent, as suggested by transcriptomic analyses, which indicate that different strains may express varying levels of virulence factors, such as cysteine proteases.^[29]^ This strain variability could explain why some individuals remain asymptomatic despite being infected, while others develop symptoms. Nonetheless, further research is needed to explore this possibility and to determine whether specific strains are more likely to cause disease.

The present results suggest that RT-PCR may detect low parasite loads, which could explain the absence of symptoms or minor symptoms, as occurs with other parasites, such as *Giardia duodenalis*.^[30]^ In the asymptomatic group of our sample, the parasite was detected at very low loads (<1 trophozoite × field) and confirmed by RT-PCR, and was significant at 47.36% (27/57). In the prevalence study of *D fragilis* in schoolchildren by Ögren et al in Sweden, RT-PCR allowed the detection of 20% more *D fragilis* in symptomatic patients compared to LM.^[31]^

In the study of the pathogenesis of parasites in which the pathogenicity has been questioned, the role of the intestinal microbiota has been postulated as a differential factor that can justify clinical manifestations. Regarding *D fragilis*, Van Kalleveen et al described no differences in the composition and diversity of the microbiota between *D fragilis* patients with diarrhea and healthy children and those with *D fragilis* with diarrhea.^[32]^

The second conclusion of this study is that the prevalence of symptomatic *D fragilis* infection is higher in pediatric patients. This has previously been reported in multiple prevalence studies, but the exact cause is unknown.^[23,33]^ A possible cause might be the presence of a symptomatic primary infection of the parasite that induces protective immunity. Additionally, children’s hygiene habits and behavioral factors, such as inadequate handwashing and frequent hand-to-mouth contact, may contribute to a higher transmission rate. Moreover, the close physical contact among children in schools and daycare settings facilitates the spread of *D fragilis*, increasing their risk of infection compared to adults.^[31,34]^

The frequency of the presentation of symptoms in our cases was similar to that of previous studies on the clinical manifestations of *D fragilis*.^[12,23]^ The proportion of positive cohabitants within household units was also similar to that of previous studies, raising questions about the role of asymptomatic carriers in perpetuating the epidemiological cycle of infection.^[24,25]^ Besides parasite load, other host-dependent factors, such as intestinal mucosal immune mechanisms, innate immune response, adaptive immune response through antibody production, or specific cellular immunity by macrophages and cytotoxic T cells, may influence the pathogenicity of an organism, since this is a dynamic process that depends on host-microorganism interaction.^[35]^

In light of our results, f-CP could be a marker of *D fragilis* pathogenicity, although the lack of statistical power in our study does not allow confirmation of this suggestion. The only study that specifically investigated f-CP levels in relation to *D fragilis* infection was by Aykur et al, who demonstrated that *D fragilis* patients had significantly higher f-CP levels compared to non-infected individuals.^[36]^ The pathophysiology of this parasite in the colon and the changes it produces at the histological level remain unclear, and thus, it is unknown if f-CP, which reflects neutrophil activity in the intestinal lumen and intestinal mucosal permeability, could be a useful biomarker.^[12]^ Salman et al studied the relationship between f-CP levels and multiple protozoa, finding that while *Entamoeba histolytica* and *Giardia lamblia* were associated with elevated f-CP levels, *B. hominis*, *Chilomastix mesnilli*, and *D fragilis* were associated with low levels.^[37]^

Interestingly, our study found no significant association between RT-PCR Ct values and symptom presentation. Ct values, which indicate the quantity of parasitic DNA in a sample, have been used in other gastrointestinal infections to correlate parasite load with clinical outcomes. However, in the case of *D fragilis*, Ct values did not appear to accurately reflect the parasite load.^[38]^ In our sample, the Ct values of the control group were 35, which is at the threshold of reliability, further complicating the interpretation of parasite load.^[39]^ This may be attributed to the inherent limitations of RT-PCR in accurately quantifying parasite load, such as the high dilution of samples, the non-homogeneous nature of fecal material, or the type of sampling method used.^[40]^

Regarding the study’s limitations, as a study analyzing microbiological samples, the variability related to the characteristics of intermittent parasite elimination and the inter-operator variability in the observation of the samples for microbiological analysis were factors influencing the results. Additionally, the absence of a standardized scale to assess symptom severity limits the ability to establish a more precise correlation with parasite load. The type of strain was also not evaluated, although previous studies have shown type 1 to be the prevalent strain.^[10,41]^

The results of this study demonstrate that parasite load measured by LM is positively correlated with clinical symptoms. The weak correlation between clinical symptomatology and the diagnosis of *D fragilis* by RT-PCR could be explained by the detection of extremely low parasite loads with minimal physiopathological impact. Another possible explanation is that the nature of the amplification technique itself may result in initial inoculum differences of less than one logarithmic unit – differences that could be clinically significant – failing to produce statistically distinct Ct values.

In conclusion, our study demonstrates a relationship between parasite load measured by LM and the presence of gastrointestinal symptoms in *D fragilis* infected individuals, highlighting the need to consider *D fragilis* as a possible etiological factor of gastrointestinal symptoms. This reinforces the idea of the pathogenic capacity of the parasite. Additionally, our results emphasize the importance of evaluating parasite load in future research on the pathogenicity of *D fragilis.*

## Acknowledgments

We would like to thank the laboratory technicians at the Microbiology Service of CATLAB: Erika Méndez Almansa, Pilar Arjona Camacho, Sandra Mascort Martí, Mireia Ribes Sole, Juani Ribas Ruiz, Montserrat Velasco Gálvez, Núria Rabaza Garrido, and Eva Bernadó Cardús for their great professionalism and technical skill.

The authors acknowledge all of the persons involved in the Dienta-Know Study Group: Anna Abugatas, Ignacio Aguilar, Cristina Alba, Enric Arroyo, Georgina Artigas, Núria Barriendos, Elisenda Benasco, Àgata Camps, Gerard Carreras, Olga Casado, Michelle de Luca, Gabriela Dodino, Alberto Galdámez, Christian Garavito, Mónica García, Romina Ginné, Raquel Hernández, Beatriz Joven, Mazen Karaki, Rabee Kazan, Beatriz Lorenzo, Laura Martí, Xavi Martínez, Sílvia Martin-Urda, Àngels Mate, Silvia Mercado, Francesc Molina, Montse Muñiz, Jéssica Ortiz, Mauricio Pérez, Josefa Pérez, Marta Pozuelo, Linda Rivera, Mar Robert, Esther Rubio, Sandra Sabarich, Xavier Salvia, Nuria Serrano, Andrea Torrabadella, Silvia Urraca, Oscar Varderi, Elba Zurdo.

## Author contributions

**Conceptualization:** Ander Burgaña Agoües, Rosa Abellana Sangra, Mónica Ballestero-Téllez, Mireia Rajadell-Guiu, Marc Garreta-Esteban, Tomàs M. Perez-Porcuna.

**Data curation:** Ander Burgaña Agoües, Rosa Abellana Sangra, Tomàs M. Perez-Porcuna.

**Formal analysis:** Ander Burgaña Agoües, Rosa Abellana Sangra, Tomàs M. Perez-Porcuna.

**Funding acquisition:** Ander Burgaña Agoües, Rosa Abellana Sangra, Tomàs M. Perez-Porcuna.

**Investigation:** Ander Burgaña Agoües, Rosa Abellana Sangra, Mónica Ballestero-Téllez, Mireia Rajadell-Guiu, Marc Garreta-Esteban, Dienta-Know Study Group, Tomàs M. Perez-Porcuna.

**Methodology:** Ander Burgaña Agoües, Rosa Abellana Sangra, Mónica Ballestero-Téllez, Mireia Rajadell-Guiu, Marc Garreta-Esteban, Tomàs M. Perez-Porcuna.

**Project administration:** Ander Burgaña Agoües, Rosa Abellana Sangra, Tomàs M. Perez-Porcuna.

**Resources:** Ander Burgaña Agoües, Rosa Abellana Sangra, Mónica Ballestero-Téllez, Mireia Rajadell-Guiu, Marc Garreta-Esteban, Tomàs M. Perez-Porcuna.

**Software:** Ander Burgaña Agoües, Rosa Abellana Sangra, Tomàs M. Perez-Porcuna.

**Supervision:** Ander Burgaña Agoües, Rosa Abellana Sangra, Tomàs M. Perez-Porcuna.

**Validation:** Ander Burgaña Agoües, Rosa Abellana Sangra, Mónica Ballestero-Téllez, Mireia Rajadell-Guiu, Marc Garreta-Esteban, Tomàs M. Perez-Porcuna.

**Visualization:** Ander Burgaña Agoües, Rosa Abellana Sangra, Tomàs M. Perez-Porcuna.

**Writing – original draft:** Ander Burgaña Agoües, Rosa Abellana Sangra, Mónica Ballestero-Téllez, Mireia Rajadell-Guiu, Marc Garreta-Esteban, Dienta-Know Study Group, Tomàs M. Perez-Porcuna.

**Writing – review & editing:** Ander Burgaña Agoües, Rosa Abellana Sangra, Mónica Ballestero-Téllez, Mireia Rajadell-Guiu, Marc Garreta-Esteban.
